# Supplementary material for: Seroprevalence and factors associated with hepatitis B virus exposure in the incarcerated population from southern Brazil
Source: PLoS One. 2022 Nov 22;17(11):e0278029. doi: 10.1371/journal.pone.0278029 (PMC9681091; doi:10.1371/journal.pone.0278029)
Supplement: Attachment — Submitted filename: Rebbutal 4.docx [file pone.0278029.s001.docx]

Francisco Beltrão, November 04, 2022

Dear Dr Jason T. Blackard,

We are pleased to send you the requested information regarding the “PONE-D-22-06352” in this new cover letter. In summary, we were asked to perform minor reviews that are detailed below. We would like to anticipate that all the suggestions were accepted.

**1^st^ request (Editor).**

Please review your reference list to ensure that it is complete and correct. If you have cited papers that have been retracted, please include the rationale for doing so in the manuscript text, or remove these references and replace them with relevant current references. Any changes to the reference list should be mentioned in the rebuttal letter that accompanies your revised manuscript. If you need to cite a retracted article, indicate the article’s retracted status in the References list and also include a citation and full reference for the retraction notice.

**Response to the 1^st^ request.**

Dear Dr. Jason, we revised all the references. There were no retracted papers cited in our study, only two references in which there were errors regarding authorship naming (i.e., Refence number 14 and Reference number 32) and one correction (errata) for Reference number 35.

As references 14 and 32 were already cited correctly, we kept these in the text. However, considering that reference number 35 contained substantial corrections, it is somewhat “old” and does not add significant evidence to our manuscript, we decided to exclude it.

We adjusted the citations in our text, and you can clearly see the changes in the file “Revised Manuscript with Track Changes”. Hyperlinks were all checked and minor corrections were performed in the reference list.

For your information, here are the reasons given by the authors for their corrections (ref. 14 and 32) and for the errata (ref. 35).

Reference 14:

*February 27, 2019: Minor Correction: Fu Hsiung Su's affiliations of “5, 7, 8, 9” should have included affiliation 3 (Department of Family Medicine, Taipei Medical University Hospital, Taipei City, Taiwan). In the Funding and Grant Disclosures, “Taipei Medical Hospital” should have been “Taipei Medical University Hospital”.*

Link: <https://peerj.com/articles/4297/>

Reference 32:

*Correction added on 2 April 2019, after first online publication: This article has been updated to correct the name of the author from ‘John Kaldo’ to ‘John Kaldor’. The second line of the ‘Results’ section of the Abstract has also been corrected to read ‘Hepatitis B’ instead of ‘Hepatitis C’, as indicated by the symbol ^ᶺ^.*

Link: <https://onlinelibrary.wiley.com/doi/full/10.1111/1753-6405.12870>

Reference 35 (excluded in the revised manuscript):

*In the MMWR Recommendations and Reports, "Prevention and Control of Infections with Hepatitis Viruses in Correctional Settings," published on January 24, 2003, an error occurred on page 4 in the second sentence of the paragraph under Occupational Exposures. The sentence should read, "Occupational transmission of HBV infection among hospital-based workers has been linked to percutaneous and mucous membrane exposures, and HCV infection has been primarily associated with percutaneous exposure." On page 12, in Box 6, the fourth item under Type of Exposure should read, "Household (e.g., cell or dormitory) contact --- to person with chronic HBV infection." On page 2, errors occurred in Table 1, and on page 20, errors occurred in Table 5.*

Link: <https://www.cdc.gov/mmwr/preview/mmwrhtml/mm5210a9.htm>

**2^nd^ request (Reviewer #1).**

The authors have satisfactorily addressed all the reviewer's comments and no further revisions. The editor should correct the typographical error on the abstract -Line 10 because it looks like a word is missing. Otherwise, the manuscript should be accepted for publication in PlosOne.

**Response to the 2^nd^ request.** We thank the reviewer for this remark. Indeed, “HBV infection” was missing from the text. We corrected this issue.

**3^rd^ request (Reviewer #2).**

1. The introduction is too long. Lines 90-115, 116-127 may be shortened and portions used while discussing the results. For examples, lines 260 to 266 seems to be a repetition of part of 104-115.

2. I think a better title will be "exposure" not "infection" with HBV. anyone with anti-HBs has recovered so cannot be infected. (see also line 182; antigens were also tested for)

3. Line 250 HBV includes virus (saying HBV virus is therefore a repetition).

**Response to the 3^rd^ request.** All these comments are extremely helpful. In the revised file, the reviewer and the editor will note that we: 1) shortened the introduction while also using some portions in the discussion; 2) revised the title and, when appropriated, substituted the word “infection” for exposure in the main text; and 3) deleted “virus” after HBV, hence avoiding repetition. All these changes are clearly marked using the “track changes” option and correspond to the sections of the text mentioned by the reviewer 2.

Finally, we followed the final instructions and removed our previous manuscript files and uploaded only this new Cover Letter. If you need any further information, please, do let us know. Once again, many thanks for your assistance.

Yours faithfully,

The authors
